# Supplementary material for: Molecular Identification of Fungal Species through Multiplex-qPCR to Determine Candidal Vulvovaginitis and Antifungal Susceptibility
Source: J Fungi (Basel). 2023 Nov 27;9(12):1145. doi: 10.3390/jof9121145 (PMC10744653; doi:10.3390/jof9121145)
Supplement: Supplementary file 1 [file jof-09-01145-s001.zip › jof-2709212-supplementary.pdf]

Supplementary material:

Table S1. Nucleotide sequences of primers for gene expression analysis by RT-qPCR (upper panel), and amplification and sequencing of *C. albicans* resistance-associated gene target region (lower panel).

**Primer**	**Sequence (5´-3´)**	**Reference**	
**RT-qPCR**			
CDR1-Fw	ATTCTAAGATGTCGTCGCAAGATG	[16]	
CDR1-Rv	AGTTCTGGCTAAATTCTGAATGTTTTC		
CDR2-Fw	TAGTCCATTCAACGGCAACATT	[16]	
CDR2-Rv	CACCCAGTATTTGGCATTGAAA		
MDR1-Fw	ACATAAATACTTTGCCCATCCAGAA	[16]	
MDR1-Rv	AAGAGTTGGTTTGTAATCGGCTAAA		
ACT1-Fw	ACGGTGAAGAAGTTGCTGCTTTAGTT	[16]	
ACT1-Rv	CGTCGTCACCGGCAAAA		
PMA1-Fw	TTGAAGATGACCACCCAATCC	[27]	
PMA1-Rv	GAAACCTCTGGAAGCAAATTCG		
**Amplification and sequencing**			
**Primer**	**Sequence (5´-3´)**	**Annealing (°C)**	**Reference**
Erg11-F2	GGGTTCCATTTGTTTACAACTTAGT	57	This study
Erg11-R1	GCAGCATCACGTCTCCAATAA		
Erg11-F3	TGACCGTTCATTTGCTCAACTA	57	This study
Erg11-R4	GATTTCTGCTGGTTCAGTAGGT		
Zinc2-1123	GATGCCAACGAATTATTGA	63	[29]
Tac1-Rv3-461	TGGTAGTGACATCGTTGGTATTG		This study
Tac1-Fw3-673	ACCTCAGTTCAAGCAAGTACTG	59	This study
Tac1-Rv2-980	CCTTTGATAGGAAAAAATATATGAAAC		
Upc2-Fw	GGCCATGCGGATAATGAGA	57	This study
Upc2-Rv	ATTACTGGTAAGGACGCTTGG		
Mrr1-F-335	GAGACTTTAGAAGAGTGAATCA	58	This study
Mrr1-R-381	TGTCATAGGGAACAACATCAT		
Mrr1-F-381	TCAATGATGTTGTTCCCTATGA	58	This study
Mrr1-R-683	CGTCTCGATACGCTAAGAA		
Mrr1-F-803	AAATCATTCTTGGTGTCAGTAT	58	This study
Mrr1-R-1037	AAAGGTGTATTGCCATAGTAA		
Mrr2-1F	GCAGAAGCGAGGGAACTTGAAA	58	[30]
Mrr2-1R	AGCACGGAGTGTGTCGTAGGAA		
Mrr2-2F	TGATCCCCATCATAGACGAAAC		
Mrr2-2R	TAGGTCCCTTGAATAAGTAGAGCG		
Mrr2-3F	AGTAGAAACCAAACTCCAAGCC		
Mrr2-3R	CGAAACTTCTGCCATCCTCAAT		

Table S2. Collection of microorganisms tested by multiplex qPCR.

***Candida* spp**	**Other yeasts**
Candida albicans (NCPF 3153; NCPF 3156; ATCC 64124; ATCC 64550; ATCC MYA 2856)	*Saccharomyces cerevisiae* (ATCC 1678)
Candida dubliniensis (NCPF 3949; NCPF 3108; CECT 11473; UPV 00-130; UPV 05-198; UPV 05-199; UPV 05-200)	*Magnusiomyces capitata* (IHEM 5665; IHEM 5666; IHEM 6803)
Candida africana (ATCC MYA 2669)	*Rhodotorula mucilaginosa* (UPV 03-307)
Candida parapsilosis (NCPF 3104; ATCC 22019)	*Cryptococcus neoformans* (ATCC 90113)
Candida orthopsilosis (ATCC 96136; ATCC 96141)	**Filamentous fungi**
Candida metapsilosis (ATCC 96143; ATCC 96144; UPV 13-078)	*Aspergillus fumigatus* (Af-293)
Candida tropicalis (NCPF 3111)	*Lomentospora prolificans* (ATCC 64913)
Candida glabrata (NCPF 3203)	**Bacteria species**
Candida nivariensis (CBS 9989)	*Staphylococcus aureus* (CECT 435)
Candida bracarensis (NCYC 3133)	*Streptococcus viridans* (CECT 804)
Candida guilliermondii (NCPF 3099)	*Streptococcus pyogenes* (CECT 985)
Candida krusei (ATCC 6258)	*Streptococcus pneumoniae* (CECT 993)
Candida lipolytica (UPV 12-097)	*Escherichia coli* (CECT 434; INVαF*)
	Klebsiella pneumoniae (CECT 144)
	Pseudomonas aeruginosa (CECT 108)
	Proteus mirabilis (CECT 4168)
	Gardnerella vaginalis (ATCC 14018)

Note. The strain reference numbers for different collections appear in brackets: ATCC=American Type Culture Collection; CBS=Centraalbureau voor Schimmelcultures; CECT=Colección Española de Cultivos Tipo; IHEM=Belgian Co-ordinated Collections of Micro-organisms; NCPF=National Collection of Pathogenic Fungi; NCYC=National Collection of Yeast Cultures; UPV=Collection from the University of the Basque Country (UPV/EHU).
*Invitrogen^{TM}.

Table S3. Specific amplification results of the designed probes for cryptic species of *Candida albicans, Candida glabrata* and *Candida parapsilosis*.

	Probes (Ct ± SD)[b]				
**Species and strains[a]**	**Calb**	**Cgla**	**Cpar3**	**Calb2**	**Cgla2**
C. albicans NCPF 3153	14.66 ± 0.021	ND	ND	14.21 ± 0.007	ND
C. dubliniensis NCPF 3949	17.28 ± 0.024	ND	ND	-	ND
C. africana ATCC MYA 2669	15.49 ± 0.25	ND	ND	15.61 ± 0.31	ND
C. glabrata NCPF 3203	ND	13.36 ± 0.0022	ND	ND	12.92 ± 0.004
C. nivariensis CBS 9989	ND	14.85 ± 0.042	ND	ND	18.23 ± 7.05
C. bracarensis NCYC 3133	ND	18.78 ± 0.2	ND	ND	18.35 ± 1.28
C. parapsilosis NCPF 3104	ND	ND	15.27 ± 0.27	ND	ND
C. orthopsilosis ATCC 96141	ND	ND	-	ND	ND
C. metapsilosis ATCC 96144	ND	ND	-	ND	ND

[a]ATCC=American Type Culture Collection; CBS=Centraalbureau voor Schimmelcultures; NCPF=National Collection of Pathogenic Fungi.
[b]The mean Ct and SD values in 3 replicates.
(-) No detection. ND: not determined

Table S4. Detection of yeasts: Comparison of vaginal swab samples (n = 129) tested in the multiplex qPCR assay with the Calb probe[a], and culture on chromogenic medium (Condalab, Madrid, Spain).

	**Culture**		
	**Positive**	**Negative**	**Total**
**Multiplex qPCR**			
**Positive**	74	5	79
**Negative**	7	43	50
**Total**	81	48	129

[a]cut-of value Ct of 30 cycles.

Table S5. In vitro antifungal susceptibility of 78 *Candida albicans* vulvovaginal isolates as determined by the CLSI method.

	24 h			48 h		
	**MIC_{50}**	**MIC_{90}**	**Range**	**MIC_{50}**	**MIC_{90}**	**Range**
**Clotrimazole**	0.03	0.06	0.03-0.25	0.12	0.25	0.03-0.5
**Fluconazole**	0.12	0.25	0.12-4	0.25	1	0.12-8

Table S6. In vitro antifungal susceptibility of 3 *Candida albicans* isolates showing reduced susceptibility to fluconazol and/or clotrimazole, as determined by Sensititre YeastOne (Trek Diagnostic System, East Grinstead, UK) microdilution method.

**Isolates**	**Be-113**		**Be-114**		**Be-129**	
	MIC (µg/ml)					
	24h	48h	24h	48h	24h	48h
CAS	0.25	0.25	0.06	0.06	0.12	0.12
MFG	0.015	0.03	0.015	0.015	0.015	0.03
AFG	0.015	0.06	0.015	0.015	0.015	0.015
AMB	0.015	0.25	0.5	1	0.5	1
5FC	0.5	2	0.06	0.12	0.12	0.25
VRC	0.5*	**8**	0.015	**8**	**1**	**2**
POS	**8**	**8**	0.015	**8**	0.06	**0.12**
ITC	**16**	**16**	0.03	**16**	0.25*	0.5*
FLC	4*	**256**	0.25	**256**	**8**	**16**

CAS=Caspofungin; MFG=Micafungin; AFG=Anidulafungin; AMB=Amphotericin B; 5FC=Flucytosine; VRC=Voriconazole; POS=Posaconazole; ITC=Itraconazole; FLC=Fluconazole.
*MIC values categorized as susceptible-dose-dependent, resistant values are highlighted in bold
